# 

**Published:** 1872-02

**Authors:** 


### INDEX OF SUBJECTS.

### ORIGINAL COMMUNICATIONS.

Annual Address before the Medical Society of North Carolina.................. 65
On the Employment of Taxis in Strangulated Hernia.................................. 83
Foreign Correspondence.................................................................. 88

### EXTRACTS AND GLEANINGS.

Sovereign Anti-Bilious Pill—Cancrum Oris, 94; Erysipelas 95; Chron-Ostitis—Amenorrhœa Successfully Treated by Electricity—Aural Polyp 96; Cerebro Spinal Meningitis, 97; Amaurosis—Broncho Pneumonia, 98; Typhoid Fever with Cappillary Bronchitis, 99; Remedy for Dandruff—Chronic Cystitis—Cure for Corns—Chloroform and Glycerine, 100; Formula in Local Use—Hydrate of Chloral, 101; Absence of Vagina and Uterus—Pills of Creosote—Pills of Carbolic Acid—A New Excipient for Pills, 102; Dimness of Vision, 103, Fissured Nipples—Scarlet Fever—Perchloride of Iron in Post partem Hemorrhage—Ovariotomy, 104; Vaccine Virus in Glycerine—Chlorate of Potassa in Dysentery, 105; Chronic Enlargement of Prostrate Gland—Spinal Irritation—Sulpho-Carbolate of Soda in Scarlatina, 106; Suppurative Hepatitis—Acid Dyspepsia—Calabar Bean in Excitement of Cerebro-Spinal Nervous System, 107; Permetritis—A Case of Glanders in a Man, 108; Charcoal in Ulcer of Stomach—Aconite Productive of Uterine Irritation—Monobromized Camphor Cotton Root in Menstrual Suppression and Dysmenorrhœ, 109; Podophyllin—Cotton Root (Gossypium) as an Antidote—Semenal Emissions, 110; Macrotys Racemosa, 111; Diagnosis of Bright's Disease—Vapor of Ammonia in the Treatment of Hooping-Cough, 112; Treatment of Chlorosis—Anhydrous Glycerine, 113.

### EDITORIALS, REVIEWS, ETC.

Our Thanks—Dr. Wm. A. B. Norcom.................................................. 124
Dr. A. W. Calhoun—Dr. C. C. F. Gay—Correction Subscribers, Take Notice..... 115
Editorial and Miscellaneous Correspondence........................................ 116
Items................................................................................... 123
Statistics of the Medical Profession in the United States......................... 126
Advertising Department.................................................................. 127

